# Intranasal *versus* intratracheal exposure to lipopolysaccharides in a murine model of acute respiratory distress syndrome

**DOI:** 10.1038/s41598-021-87462-x

**Published:** 2021-04-08

**Authors:** Fatemeh Khadangi, Anne-Sophie Forgues, Sophie Tremblay-Pitre, Alexis Dufour-Mailhot, Cyndi Henry, Magali Boucher, Marie-Josée Beaulieu, Mathieu Morissette, Liah Fereydoonzad, David Brunet, Annette Robichaud, Ynuk Bossé

**Affiliations:** 1 Institut Universitaire de Cardiologie et de Pneumologie de Québec – Université Laval, Pavillon Mallet, M2694, 2725, chemin Sainte-Foy, Quebec, QC G1V 4G5 Canada; 2 SCIREQ – Scientific Respiratory Equipment Inc., Montreal, Canada

**Keywords:** Experimental models of disease, Preclinical research

## Abstract

Due to frequent and often severe lung affections caused by COVID-19, murine models of acute respiratory distress syndrome (ARDS) are increasingly used in experimental lung research. The one induced by a single lipopolysaccharide (LPS) exposure is practical. However, whether it is preferable to administer LPS intranasally or intratracheally remains an open question. Herein, female C57Bl/6 J mice were exposed intranasally or intratracheally to one dose of either saline or 3 mg/kg of LPS. They were studied 24 h later. The groups treated with LPS, either intranasally or intratracheally, exhibited a pronounced neutrophilic inflammation, signs of lung tissue damage and protein extravasation into the alveoli, and mild lung dysfunction. The magnitude of the response was generally not different between groups exposed intranasally *versus* intratracheally. However, the variability of some the responses was smaller in the LPS-treated groups exposed intranasally *versus* intratracheally. Notably, the saline-treated mice exposed intratracheally demonstrated a mild neutrophilic inflammation and alterations of the airway epithelium. We conclude that an intranasal exposure is as effective as an intratracheal exposure in a murine model of ARDS induced by LPS. Additionally, the groups exposed intranasally demonstrated less variability in the responses to LPS and less complications associated with the sham procedure.

## Introduction

Within the last months, the severe acute respiratory syndrome-coronavirus 2 (SARS-CoV-2) has been infecting between 3 and 8 hundred thousand of new individuals each day^1^. Five percent of SARS-CoV-2-infected patients develop a critical form of the disease that progresses to acute respiratory distress syndrome (ARDS)^2,3^. Among them, approximately half are dying^2–4^.

Animal models will be of utmost importance in expediting the development of therapeutics for COVID-19. Since a fraction of SARS-CoV-2-infected patients degenerates into ARDS^2,3^, animal models of ARDS will be increasingly used in experimental lung research. There is obviously no animal model that fully recapitulates the whole spectrum of features present in human ARDS^5,6^. However, numerous animal models exhibit various typical features relevant to ARDS^5,6^. The optimal choice of animal model ultimately depends on the scientific question being addressed^7^.

Among the animal species used to model ARDS^5,6^, mice are particularly convenient because they are fast breeders, they are relatively low-cost and a vast array of molecular tools and equipment are available for mechanistic studies. One of the widely utilized murine models of ARDS is the one induced by a single lipopolysaccharide (LPS) exposure^6,8^. The procedures to establish the model are relatively straightforward and require minimal mouse handling skills. The fact that it is aseptic is another practical advantage. This means that it merely requires Biosafety level 1, which is accredited to many laboratories. Finally, the outcomes can be measured with relative ease and represent relevant features of human ARDS, including neutrophilic inflammation, signs of damage to the alveolar capillary barrier, lung tissue injury, and physiological dysfunction^6,9^. It is therefore a good model for both pragmatic and scientific reasons.

**Figure 1:**
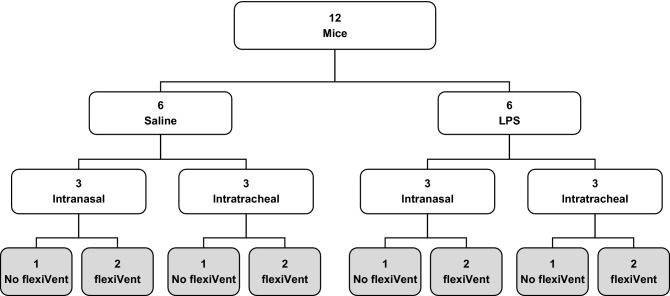
Schematic of the experimental protocol. On day 1, the mice were assigned to one of four experimental groups: 1- saline delivered intranasally; 2- saline delivered intratracheally; 3- LPS delivered intranasally; or 4- LPS delivered intratracheally. On day 2 (in gray), exactly 24 h after saline or LPS exposure, the mice were tested. Respiratory mechanics was assessed with the flexiVent on 2 mice in each group. The bronchoalveolar lavages, the lung wet-to-dry weight ratio, and the histology on lung slices were performed on all mice. The contractile assays with excised tracheas were performed on one mouse tested with the flexiVent and one mouse not tested with the flexiVent in each group. This protocol was repeated 6 times to reach the sample sizes shown in Table 1.

Yet, the procedures to set out the murine model of ARDS induced by LPS are not fully standardized and some specific technicalities were shown to differ among previous users. One such unresolved technical question is to whether LPS should be administered intranasally or intratracheally. In the present study, we compare the intranasal (IN) and intratracheal (IT) routes of administration in terms of both the magnitude and the variability of the response to LPS for several outcomes that are traditionally used to assess ARDS in animal models^9^ (Fig. 1).

## Results

### Cellular inflammation

**Figure 2:**
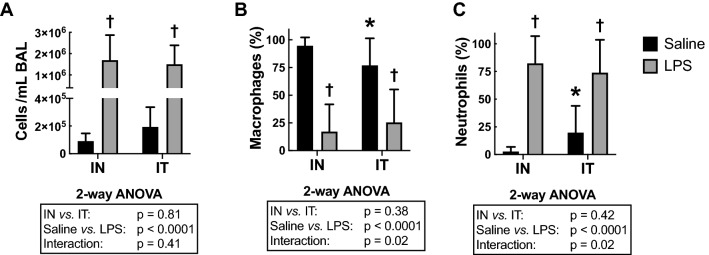
Cellular lung inflammation in the bronchoalveolar lavages. (**A**) Total cells per mL of BAL, and the percentage of (**B**) macrophages and (**C**) neutrophils in BAL are depicted for mice treated with either saline (black) or LPS (grey) through either the intranasal (IN) or the intratracheal (IT) route. The results of the two-way ANOVA are shown at the bottom of each graph. *Indicates statistically significant from the IN-exposed group treated with saline. †Indicates statistically significant from the group treated with saline exposed through the same route. n = 18 per group and the data shown are means ± SD.

LPS increased cellular lung inflammation, as seen by the number of cells/mL in bronchoalveolar lavages (BAL) (*P* < 0.0001; Fig. 2A). This was mainly due to recruitment of neutrophils, as seen by a significant increase in the percentage of neutrophils (*P* < 0.0001; Fig. 2C) and a corresponding decrease in the percentage of macrophages (*P* < 0.0001; Fig. 2B). Very few eosinophils and lymphocytes were detected in each of the four groups (data not shown).

The route of LPS administration did not affect the number of cells/mL in BAL. However, there was a significant interaction between treatment (saline *vs.* LPS) and its route of administration for both the percentage of neutrophils (*P* = 0.02) and macrophages (*P* = 0.02). This was mainly driven by an increased percentage of neutrophils, and a corresponding decrease in the percentage of macrophages, in the IT-exposed group *versus* the IN-exposed group treated with saline (Fig. 2B and C). Post-hoc analysis revealed that both the percentage of neutrophils (*P* = 0.05; 19.8 ± 24.2 *vs.* 2.7 ± 4.3%) and macrophages (*P* = 0.04; 76.9 ± 24.5 *vs.* 94.8 ± 7.5%) were different in the IT-exposed group *versus* the IN-exposed group treated with saline. Although the IT-exposed group treated with saline also demonstrated a numerically increased number of cells/mL in BAL compared to the IN-exposed group treated with saline (1.9 × 10^5^ ± 1.4 × 10^5^ *vs.* 9.1 × 10^4^ ± 5.4 × 10^4^), this did not reach statistical significance. The variability in the number of cells/mL in BAL, the percentage of neutrophils and the percentage of macrophages was also increased by IT *versus* IN in mice treated with saline, but not in mice treated with LPS.

**Figure 3:**
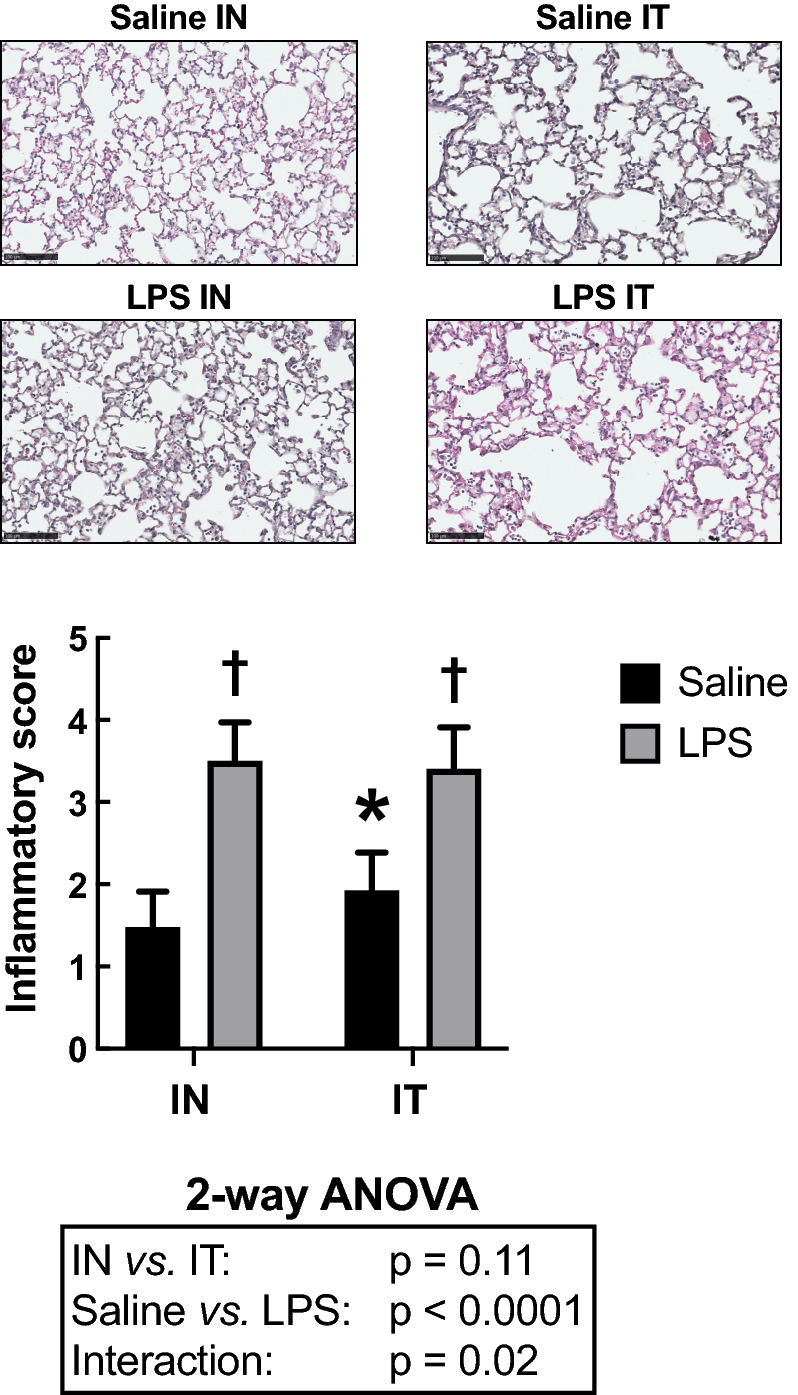
Cellular inflammatory infiltrates into the parenchymal tissue. The upper panels are representative lung sections derived from mice treated with either saline (top) or LPS (bottom) through either the intranasal (IN) or the intratracheal (IT) route. The bar graph below depicts the mean inflammatory score in each group. Scale bars are 100 µm. The results of the two-way ANOVA are shown at the bottom of the graph. *Indicates statistically significant from the IN-exposed group treated with saline. †Indicates statistically significant from the group treated with saline exposed through the same route. n = 18 per group and the data shown are means ± SD.

To confirm the presence of inflammation within the lung parenchyma, tissue infiltration with inflammatory cells was quantified by histology (Fig. 3). As per the BAL, LPS significantly increased the inflammatory score within the parenchyma (*P* < 0.0001). The route of LPS administration did not significantly affect the inflammatory score. However, there was a significant interaction between the treatment (saline *vs.* LPS) and its route of administration (*P* = 0.02). Post-hoc analysis revealed that the inflammatory score in saline-treated mice was greater in the IT-exposed group *versus* the IN-exposed group (*P* = 0.01; 1.9 ± 0.5 *vs.* 1.5 ± 0.4). The variability did not differ between groups.

**Figure 4:**
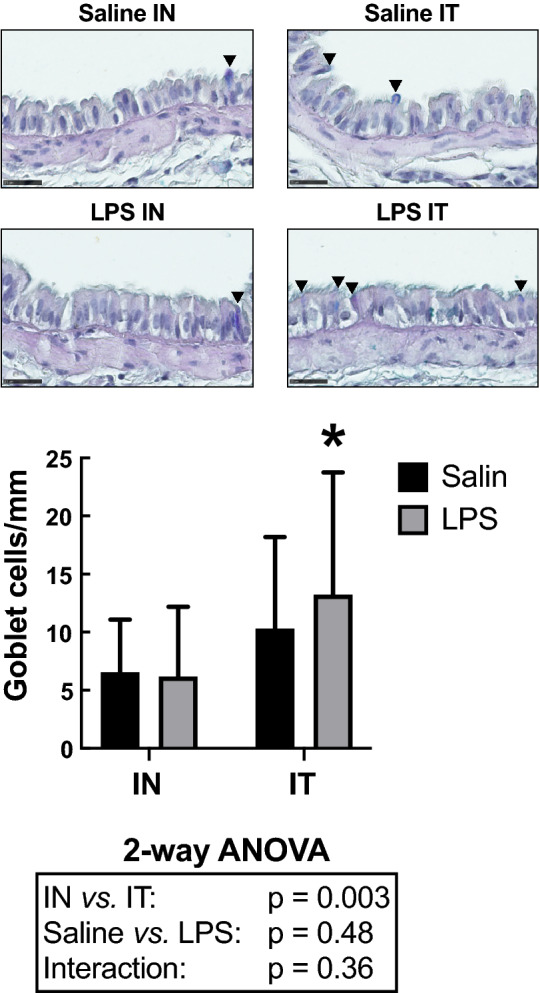
Number of goblet cells per mm of basement membrane. The upper panels are representative lung sections derived from mice treated with either saline (top) or LPS (bottom) through either the intranasal (IN) or the intratracheal (IT) route. The bar graph below depicts the mean in each group. Scale bars are 25 µm. The results of the two-way ANOVA are shown at the bottom of the graph. *Indicates statistically significant from the IN-exposed group treated with LPS. n = 18 per group and the data shown are means ± SD.

The number of goblet cells lining the epithelium was also assessed as an additional histological sign of inflammation (Fig. 4). LPS did not affect counts of goblet cells (*P* = 0.48). However, goblet cell counts were significantly affected by the route of administration (*P* = 0.003). IT-exposed mice demonstrated an increased number of goblet cells compared to IN-exposed mice. Post-hoc analysis revealed that the goblet cell count in LPS-treated mice was greater in the IT-exposed group *versus* the IN-exposed group (*P* = 0.01). IT also significantly increased variability compared to IN (*P* = 0.006).

### Protein extravasation in the alveoli

**Figure 5:**
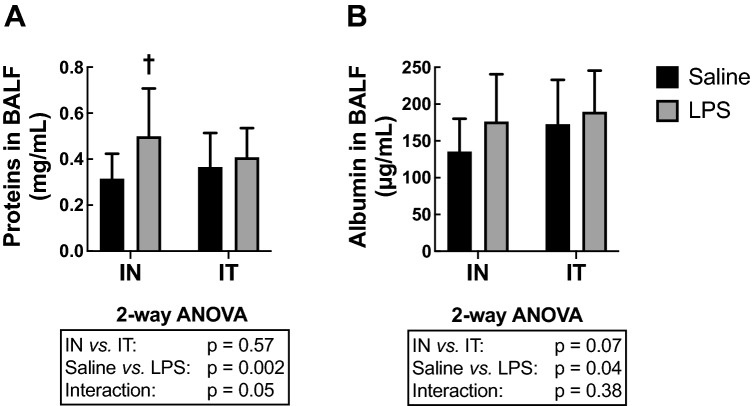
Protein extravasation into the alveoli. The concentration of (**A**) total proteins and (**B**) albumin in bronchoalveolar lavage fluid (BALF) are depicted for mice treated with either saline (black) or LPS (grey) through either the intranasal (IN) or the intratracheal (IT) route. The results of the two-way ANOVA are shown at the bottom of each graph. †Indicates statistically significant from the group treated with saline exposed through the same route. n = 18 per group and the data shown are means ± SD.

LPS increased the concentration of total proteins in BAL fluids (BALF) (*P* = 0.002: Fig. 5A). The route of LPS administration did not affect the concentration of total proteins in BALF. However, there was a significant interaction between treatment (Saline *vs.* LPS) and the route of administration (*P* = 0.05). This is because the concentration of total proteins in saline-treated groups tended to be greater in the IT-exposed group *versus* the IN-exposed group and, inversely, the protein concentration in LPS-treated groups tended to be lower in the IT-exposed group *versus* the IN-exposed group. This interaction also explained why the effect of LPS seemed greater in the IN-exposed groups *versus* the IT-exposed groups. In fact, post hoc analyses revealed that the effect of LPS was significant in the IN-exposed groups (*P* = 0.001) but not in the IT-exposed groups (*P* = 0.65). The variability did not differ between groups.

The concentration of albumin in BALF was also assessed as an additional sign of damage to the alveolar capillary barrier (Fig. 5B). One outlier was removed from the IT-exposed group treated with saline and two outliers were removed from the IT-exposed group treated with LPS (as they were beyond two standard deviations from the mean). LPS increased the BALF concentration of albumin (*P* = 0.04) but the route of administration had no effect (*P* = 0.07). Post-hoc analysis revealed that the increase in albumin concentration caused by LPS was almost significant in the IN-exposed groups (*P* = 0.06) but not in the IT-exposed groups (*P* = 0.65). The variability did not differ between groups.

### Respiratory mechanics

**Figure 6:**
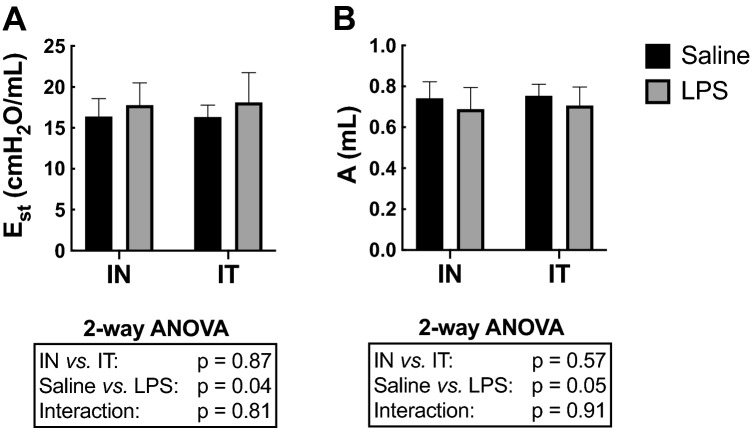
Respiratory mechanics. (**A**) The quasi-static elastance of the respiratory system (E_st_) and (**B**) A (a proxy for the inspiratory capacity) are depicted for mice treated with either saline (black) or LPS (grey) through either the intranasal (IN) or the intratracheal (IT) route. The results of the two-way ANOVA are shown at the bottom of each graph. n = 12 per group and the data shown are means ± SD.

LPS increased quasi-static elastance (E_st_) (*P* = 0.04; Fig. 6A). There was also a significant decrease in A (a proxy for the inspiratory capacity) caused by LPS (*P* = 0.05; Fig. 6B), suggesting that the increase in E_st_ was mainly due to a decrease in the accessible lung volume. All the other parameters of respiratory mechanics were not significantly affected by LPS.

The route of LPS administration did not influence the mean values of any measured parameter. However, the variability of E_st_ was significantly greater in the group exposed to LPS by IT than the other 3 groups (p = 0.04). The same trend was also observed for most parameters, but it only reached statistical significance for K, which is a volume-independent indicator of lung compliance (see methods) (*P* < 0.0001).

### Lung wet-to-dry weight ratios

The wet-to-dry ratios were not significantly affected by either LPS or its route of administration (data not shown).

### Contractility of excised tracheas

**Figure 7:**
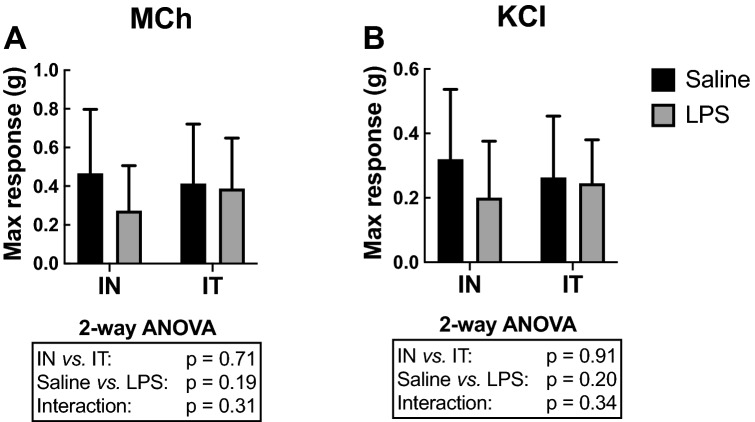
Contractility of excised tracheas. The maximal force generated by excised tracheas in response to (**A**) a cumulative concentration of methacholine (MCh) ending with 10^–4^ M or (**B**) a cumulative concentration of potassium chloride (KCl) ending with 160 mM are depicted for mice treated in vivo with either saline (black) or LPS (grey) through either the intranasal (IN) or the intratracheal (IT) route. The results of the two-way ANOVA are shown at the bottom of each graph. n = 12 per group and the data shown are means ± SD.

The maximal force generated by the tracheas in response to either methacholine or potassium chloride (KCl) was not significantly affected by either LPS or its route of administration (Fig. 7). The EC50 (the concentration causing 50% of the maximal response, an index of sensitivity) was also calculated for both methacholine and KCl and, again, no significant effect of either LPS or its route of administration was observed (data not shown). The variability also did not differ between groups for the force generated by the tracheas in response to both methacholine and KCl in terms of both the maximal response and the EC50.

## Discussion

We set out to investigate whether it is preferable to administer LPS intranasally or intratracheally in a murine model of ARDS. The conclusions are quite straightforward. Both routes of administration were able to recapitulate features of ARDS that are intended by this model. Investigators opting for this model should thus expect the same magnitude of responses to LPS by using either route of administration. However, IT exposure led to a greater inter-subject variability in some of the measured outcomes. Additionally, IT exposure in the saline-treated mice was associated with a greater percentage of neutrophils in the BAL and an increased number of goblet cells within the epithelium. Since neutrophilia is an intended feature of this model, we are tempted to recommend the use of the IN route of LPS administration to avoid this putative confounding factor.

Our results confirmed that the murine model of ARDS induced by a single exposure to LPS exhibits the four cardinal features of experimental ARDS; namely an inflammatory response, aberrant alveolar capillary barrier, histological evidence of tissue injury, and physiological dysfunction^9^. The model should thus meet its goal of accelerating experimental lung research that aims at discovering and developing improved therapeutics against ARDS. Our results also demonstrated that opting for either the IN or IT route of LPS administration does not overly influence the validity of the model.

The more obvious alteration seen in this model is cellular lung inflammation^6^. In our experiments, the number of cells per mL of BAL reaches over 1.5 million, which represented an approximately 16-fold increase compared to mice treated with saline. Most of the recruited cells (over 74%) were neutrophils. These results were confirmed by histologic assessment of tissue infiltration with inflammatory cells within the parenchyma. In comparison, the other measured outcomes were affected rather modestly by LPS. For example, many parameters of respiratory mechanics, including common ones such as respiratory system resistance (Rrs), were not significantly affected 24 h after LPS exposure. Yet, the quasi-static elastance (E_st_) of the respiratory system was significantly increased, which is a typical feature of patients with ARDS^10^. This latter increase occurred in conjunction with a decrease in the parameter A of the Salazar-Knowles’s equation, which is a proxy for the inspiratory capacity. Together, these results suggest that the increase in E_st_ was due to a loss in accessible lung volume. This loss in accessible lung volume is presumably due to pulmonary consolidations caused by alveolar engorgement secondary to the ongoing inflammation and the accompanying edema and mucus secretion. This conjecture is concordant with the increased concentration of total proteins and albumin found in the BALF, which is an indicator of protein extravasation into the alveoli and a surrogate for quantifying the amount of damage to the alveolar capillary barrier. The extent of the alveolar engorgement was mild though, as no significant difference was observed in the lung wet-to-dry weight ratios. Similarly, although the effect of LPS was significant on BALF’s albumin concentration based on a two-way ANOVA, post-hoc tests were not significant for both the IN- and the IT-exposed groups. It is possible that a longer LPS exposure or a greater LPS dose would have aggravated the damage to the alveolar capillary barrier, as well as the alveolar engorgement and the associated lung dysfunction. Importantly, other typical features of human ARDS are either very weakly expressed or completely lacking in the model described herein, such as hemorrhage, diffused alveolar damage, edema of the alveolar septa and hyalin membrane deposition^11^. It is strongly recommended to consider an alternative model to study these other specific features of ARDS^12–14^. Reviews outlining features exhibited by different models are available^6,9^.

As per the contraction of airway smooth muscle, the lack of effect of LPS on the contractile capacity of excised tracheas is consistent with a previous study^15^. In fact, similar to that latter study, we rather observed a tendency towards a decline in contractility induced by LPS^15^. It is worth mentioning though that other studies have reported either enhancing, inhibiting or no effect of LPS on airway smooth muscle contraction^16–29^. The reasons for these discrepancies will need to be sorted out.

By comparing the IN and IT routes of LPS administration, we identify two major concerns with the IT. The first concern was the greater inter-subject variability in some of the measured outcomes, especially the parameters of respiratory mechanics. Although the procedures to administer substances through IT has been described in details and can be performed with relative ease by trained research staff^30^, it still represents a greater technical challenge than IN. We thus attribute the greater inter-subject variability to technical difficulties, which may have altered the amount of LPS delivered in each mouse.

The second and more important concern was signs of inflammation in the IT-exposed mice treated with saline, evidenced by a greater percentage of neutrophils in BAL and an increased cell count of goblet cells in the epithelium. Since neutrophilia is an intended feature of the model, the neutrophilia induced by IT represents a confounding factor that may putatively decrease the differences between the saline and the LPS-treated groups. In our study, for example, this confounding factor may have contributed to the lack of significant effect of LPS on BALF total protein concentration in the IT-exposed mice (while LPS significantly increased the concentration of total proteins in the BALF of IN-exposed mice). For this reason, and the greater inter-subject variability outlined above, opting for IN over IT might be a better choice. One can also argue that IN is more suitable than IT since it recapitulates one additional feature of ARDS, namely protein extravasation into the alveoli.

In the midst of this pandemic, animal models are eagerly needed to study the pathogenicity of SARS-CoV-2 and to evaluate vaccines and therapeutics^7^. It is understood that traditional models of ARDS, including the murine model induced by LPS, may not be adequate to study COVID-19 pathogenicity. Models using viral infection represent better choices. Many species have been experimentally exposed to SARS-CoV-2, including non-human primates, pigs, dogs, cats, ferrets, ducks, chickens, hamsters and mice^7,31^. While some species support viral replication, such as non-human primates, dogs, cats, ferrets and hamsters, they all developed either none or mild pathological responses and clinical signs, suggesting different pathogenicity between species^31^. Alternative strategies are now being employed to adapt the virus to make it permissive to mice, and to use transgenic mice lacking specific immune cells or molecules rendering them more vulnerable to the virus or expressing human proteins that facilitate viral entry or replication into the cells^32–38^. Although the cause of mortality in those early models was due to high viral replication in the brain^32^, suggesting again a very different inter-species pathogenicity, the most recent models are more promising, showing respiratory system dysfunctions and typical signs of acute lung injury (ALI) and ARDS leading to mortality^39,40^.

One major shortcoming with these latter models is accessibility. The facilities for conducting such experiments necessitate high level of biosafety containment, entail high costs for infrastructure and maintenance, and require highly qualified staff and expertise. Consequently, these models are not accessible to most scientists. Obviously, this does not mean that other scientists cannot participate in this battle against COVID-19.

Many other models can be used to evaluate the efficacy of therapeutics that are designed to either prevent or reverse specific features commonly found in COVID-19. In fact, due to the large spectrum of disease severities and clinical presentations of SARS-CoV-2-infected patients, it would probably be misleading to use a single animal model. The proper choice of model rests on the scientific questions being addressed. Several animal models that are relatively simple and accessible to most scientists display one or several relevant features of COVID-19. They can thus be used to provide valuable insights. It is also important to keep in mind that for each of these relevant features, the number of molecular mechanisms for which targeting may benefit abound. In order to reduce the overall burden of COVID-19, we need to explore a horizon of molecular mechanisms as large as possible for each of these features and, for this, we need to engage the entire scientific community.

ARDS is a typical example of a feature found in a fraction of SARS-CoV-2-infected patients that can be studied in animal models. Indeed, we believe that the approach is valid for the sake of a first screen to test exploratory therapeutics. It is worth noting that the form of ARDS induced by SARS-CoV-2 is somewhat atypical. It has actually been coined ‘CARDS’ for coronavirus disease-associated ARDS^41^. Yet, CARDS presents features that are consistent with the Berlin definition of ARDS^10^. Although not measured herein, LPS exposure also causes features of microvascular lung injury^8^, which is relevant for an endothelium-avid virus such as SARS-CoV-2^41^. The fact that patients afflicted by CARDS respond similarly to conventional ARDS treatments lends further support to the notion that traditional models of ARDS can be used to assess therapeutics against CARDS^42^.

As these ARDS models are gaining in popularity, efforts of standardization of procedures are also needed. This is required to generate results that are consistent and reproducible, and that will then enable appropriate comparisons between tested drugs and different laboratories. Our study was specifically designed to address an unresolved technical question that keeps many scientists wondering. It thus fits within this overall effort of standardization of procedures. More precisely, the IN and IT routes of treatment administration were compared in a common and accessible murine model of ARDS induced by a single dose of LPS. We conclude that an IN exposure is as effective as an IT exposure. The IN exposure has the additional advantages of showing less variability in some of the responses to LPS and to avoid neutrophil recruitment and epithelial metaplasia associated with the IT procedure. We believe that our findings will enlighten many investigators who wish to test the efficacy of therapeutics on this model of ARDS. Notably, our study is also useful because the LPS model can equally be used to study other diseases, such as lung bacterial infection, or to study specific fundamental processes leading to common diseases, such as the migration and infiltration of neutrophils into a tissue. Our study will thus contribute to accelerate drug development in several diseases, including ARDS in the context of COVID-19 or otherwise.

## Methods

### Animals

Seventy-two female C57BL/6 J mice were purchased from The Jackson Laboratory (Bar Harbor, ME) at 11 weeks old. The mice were provided food and water ad libitum and were housed one week before the experiments. All procedures were approved by the Committee of Animal Care of *Université Laval* in accordance with the guidelines of the Canadian Council on Animal Care (protocol 2020–593-1) and comply with the ARRIVE guidelines (http://www.nc3rs.org.uk/page.asp?id=1357).

### Protocol

Each protocol lasted 2 days. On day one, 12 mice were assigned to one of the four experimental groups. The schematic is shown in Fig. 1. They were either treated with saline (0.9%) or LPS at 3 mg/kg (Lipopolysaccharides from Escherichia coli O55:B5, Sigma, lot number: 026M4173V). While half the mice were exposed to the treatment intranasally, the other half were exposed intratracheally.

The following describes how the IN and the IT were performed. For both routes of administration, the mouse is first sedated with isoflurane before exposure. The intranasal exposure consists at introducing the solution into one nostril. The solution of LPS is first prepared at 3 µg per µL of saline. 1 µl per gram of body weight is introduced (*e.g.* 25 µl for a mouse of 25 g) with a pipette during the inspiration (the sedated mouse breathes at a slower frequency than normal, at approximately 1 breath per second). The whole content is generally administered within 2 or 3 tidal breaths. The intratracheal exposure consists at introducing the same solution directly into the trachea. The mouse is lying supine on a platform inclined at 45º, hanging stably with an elastic rubber band around its incisors. The solution is first aspirated with a syringe into the distal end of a cannula (20G without the needle), which is then introduced into the tracheal opening. The tracheal opening is localized by identifying the vocal cords with the aid of an otoscope equipped with an adjustable operating head and a speculum that is specifically designed to perform IT in mouse (Hallowell EMC, Pittsfield, MA). Once intubated, the syringe is removed and the solution is aspirated through spontaneous inspirations of the mouse over a few tidal breaths. The aspiration of the solution also confirms that the cannula is correctly position into the trachea. The syringe is then reconnected to the cannula and a bolus of 200 µl of air is injected into the lung to ensure that the whole content is administered, which also helps breathing after the procedure because it removes the solution from the large airways. The mouse remains on the platform a few seconds after the procedure. Both the IN and the IT procedures need to be performed swiftly as the mouse usually regains consciousness in generally less than one minute.

The treatments were staggered throughout the day according to the duration of the experiments on the second day in order to ascertain that each mouse was exposed exactly 24 h. The time of the day when each experimental group was treated was also randomized to prevent circadian confounders. On day two, all the outcomes were collected. We assessed the outcomes 24 h after exposure because it corresponds to the time at which the peak influx of neutrophils in the lung is observed in this model^43^. It is also recommended that maximal lung injury should be evident within 24 h of exposure to the inciting stimulus in animal models of ARDS^9^. This is because a rapid onset is a characteristic feature of human ARDS and should ideally be simulated in animal models. In each experimental group, 2/3 of the mice underwent the measurements of respiratory mechanics with the flexiVent. The other 1/3 did not. The purpose of the latter sub-group was to determine whether measurements of respiratory mechanics with the flexiVent affect the outcomes that were subsequently measured. The bronchoalveolar lavages, the lung wet-to-dry weight ratio, and the histology on lung slices were performed on all mice. The contractile assays with excised tracheas were performed on one mouse tested with the flexiVent and one mouse not tested with the flexiVent in each group. The whole protocol was repeated on 6 occasions. The total number of mice per group is shown in Table 1.

**Table 1:** Sample size per group.

	No flexiVent		flexiVent^a^		Combined^a^	
	IN	IT	IN	IT	IN	IT
Saline	6	6	12	12	18	18
LPS	6	6	12	12	18	18

*IN* intranasal, *IT* intratracheal, *LPS* lipopolysaccharides.

^a^The only exception was the contractile assays with excised tracheas, where only half of the mice that underwent measurements with the flexiVent were tested. This reduced the sample size to 6 per group for the mice that underwent measurements with the flexiVent and to 12 per group when the mice tested or not with the flexiVent were combined.

### Respiratory mechanics

Baseline respiratory mechanics was assessed using the flexiVent (SCIREQ, Montreal, PQ, Canada)^44^. The mice were anesthetized with ketamine and xylazine at 100 and 10 mg/kg, respectively. They were then tracheotomized and connected to the flexiVent in a supine position. The ventilation was set at a breathing frequency of 150 breaths/minute, a tidal volume of 10 mL/Kg and a positive end-expiratory pressure of 3 cmH_2_O. Once the ventilation was established, the mice were paralyzed with 0.1 mg/kg of pancuronium bromide injected intramuscularly.

Baseline respiratory mechanics was measured 10 s after two deep inflations to 30 cmH_2_O, which are required for lung recruitment. Two distinct volume-perturbation maneuvers were then used to test the mechanics of the respiratory system, which are called the SnapShot-150 and the Quick Prime-3. They were both actuated twice in an alternating fashion, each being intercalated by 8 s of tidal breathing to prevent desaturation. The volume perturbation imparted by the SnapShot-150 is a single sine wave oscillation that allows one to infer values for the resistance (Rrs) and elastance (Ers) of the respiratory system based on the single-compartment model^45^. The volume perturbation imparted by the Quick Prime-3 is a composite signal constituted of 13 sine waves, all at different frequencies (spanning from 1 to 20.5 Hz), amplitudes and phases, that allows one to infer values for Newtonian resistance (R_N_), tissue damping (G) and tissue elastance (H) based on the one-phase constant model^46^. The mice were then subjected to a partial pressure–volume (P–V) maneuver. The P–V maneuver consists at inflating the lung by step increases in pressure from 3 to 30 cmH_2_O and then deflating it by step decreases back to 3 cmH_2_O. The changes in volume at the different holding (static) pressures were then plotted for both the inflating and deflating limbs of the maneuver to form the P–V loop. The data were then analyzed using the Salazar-Knowles’s equation: V = A − B*e*^-KP^, where V is volume, A is the asymptote on the volume-axis (it is a fair estimate of the inspiratory capacity but assessed in quasi-static conditions), B is the difference between A and the extrapolated volume at which pressure would cross zero, K is an exponent describing the curvature of the upper portion of the deflation limb of the P–V loop, and P is pressure. Two additional parameters were calculated from the quasi-static P–V loop, namely quasi-static elastance (E_st_) and hysteresis (*i.e.*, the area within the P–V loop). E_st_ is the reciprocal of quasi-static compliance (C_st_) and is extracted from the Salazar-Knowles equation at 5 cmH_2_O.

### Bronchoalveolar lavages

The bronchoalveolar lavages (BAL) were collected in the left lung immediately after measurements of respiratory mechanics while the mouse was still tracheally cannulated. The main right bronchus was first ligated prior to the lavages with phosphate buffered saline (PBS). This was to avoid leakage of PBS into the right lung, which would have invalidated the subsequent measurement of the right lung wet-to-dry weight ratio. The lavages were actually performed by 2 consecutive cycles of injection and aspiration of 0.25 mL of sterile PBS. The aspirated volumes were pooled and centrifuged at 500 × g during 5 min. The recovered pellet was then resuspended in 100 µl of PBS and a small fraction (10 µl) was used to estimate the total number of cells using an hemacytometer. The remaining cells were cytospun on a microscopic slide, which was then stained with modified May-Grünwald Giemsa stain (HemaStain Set, Fisher Scientific, Kalamazoo, MI) to assess percentages of macrophages, lymphocytes, neutrophils and eosinophils. The BAL supernatant was also collected and stored at -80 °C. The supernatant was used to assess the concentration of total proteins in BAL fluid (BALF). It was done using the Pierce BCA Protein Assay Kit (Thermo Fisher Scientific, Waltham, MA, USA) according to manufactured instructions. An increased protein concentration in BALF is an indicator of alterations of the alveolar capillary barrier, which is pathognomonic of ARDS^47^. Since a protein-rich edema fluid in the alveolar compartment may also stem solely from inflammation (*i.e.*, without any damage to the alveolar capillary barrier), BALF were also used to measure the expression level of albumin. It was done using a mouse albumin ELISA kit (Abcam, Cambridge, UK) according to manufactured instructions. Albumin is a high molecular weight protein predominantly found in the circulation. The presence of high concentrations of albumin in BALF confirms that the integrity of the alveolar capillary barrier was compromised.

### Lung wet-to-dry weight ratios

The right lung was used to calculate the wet-to-dry weight ratios. The four lobes were weighed immediately after excision. They were then left to dry for 5 days in an incubator set at 60 °C and weighed again.

### Contractile assays with excised tracheas

Because of the limited number of organ baths available, the trachea of only one mouse that underwent measurements with the flexiVent was tested (instead of two; see Fig. 1). Thus, 8 tracheas per protocol were tested for a total of 48 in our study. The methods were described previously^48^. Briefly, the whole trachea was excised after the BAL and immersed into Krebs solution (pH 7.4, 111.9 mM NaCl, 5.0 mM KCl, 1.0 mm KH_2_PO_4_, 2.1 mM MgSO_4_, 29.8 mM NaHCO_3_, 11.5 mM glucose, and 2.9 mM CaCl_2_). The trachea was then mounted horizontally in a 10-mL organ bath containing Krebs solution that was maintained at 37 °C. It was connected to a force transducer (Harvard Apparatus, St-Laurent, PQ, Canada) and subjected to an initial distending force of 5 mN. This resting force and the isometric force generated by the trachea in response to contractile activation were monitored at all time. Prior to any recorded measurement, the trachea was subjected to a period of conditioning, during which time it was stimulated to contract repeatedly for 5 min at 10-min intervals with 10^–5^ M of methacholine until a reproducible force was recorded.

Cumulative concentration–response curves were generated with two distinct spasmogens, namely methacholine and potassium chloride (KCl). Methacholine was added in log increments at 5-min intervals from 10^–7^ to 10^–4^ M. The concentration of KCl was doubled at 5-min intervals from 20 to 160 mM. The peak force obtained at each concentration was used in the reported data. At least 30 min were left between the two spasmogens, over which time the trachea was repeatedly washed with fresh Krebs.

### Histology on lung slices

Histology was performed on the left lung. Immediately after euthanasia, the left lung was immersed in formalin during 24 h for fixation. It was then dehydrated in 50% ethanol until further processed. The lobe was embedded in paraffin, cut transversally in 5 µm-thick sections and stained with either hematoxylin and eosin (H & E) or Alcian blue periodic acid shift (AB-PAS). The sections were then imaged with a NanoZoomer Digital scanner (Hamamastu Photonics, Bridgewater, NJ, USA). The sections stained with H & E were used to quantify tissue infiltration with inflammatory cells. Parenthetically, the accumulation of inflammatory cells in the alveolar and the interstitial spaces is considered an histological evidence of tissue injury in animal models of ARDS^9^. For each mouse, 15 non-overlapping photomicrographs (1440 × 904 pixels) of the lung parenchyma from 3 non-contiguous lung sections were blindly scored from zero (no inflammation) to 5 (very severe inflammation) by one observer. The scores from each of the 15 photomicrographs were averaged to obtain one value per mouse and the values from each mouse within one group were then compiled to obtain a mean per group. The sections stained with AB-PAS were used to count the number of goblet cells in the epithelium^49^. Three sections in each mouse were used. All airways cut transversally and with an internal diameter over 200 µm were analyzed. An average of 3.2 ± 0.9 airways per mouse were analysed. For each analyzed airway, the number of goblet cells was divided by the length of the basement membrane. The values from each of the individual airway were averaged to obtain one value per mouse and the values from each mouse within one group were then compiled to obtain a mean per group.

### Statistical analyses

Data are shown as means ± SD. Two-way ANOVAs were used to assess the effect of treatment (saline *versus* LPS), the effect of the route of administration (IN *versus* IT) and their interaction on each measured outcome. When appropriate, Sidak’s multiple comparison test was used to identify which groups significantly differ from others. Since measurements with the flexiVent had no significant effect on any of the subsequently measured outcomes, including inflammatory cells in BAL, the lung wet-to-dry weight ratios, the contractile assays with the excised tracheas and the histologic readouts, the mice tested or not with the flexiVent were pooled (Table 1). For each outcome, a Bartlett’s test was used to compare the variability between groups. All statistical analyses were performed using Prism 8 (Version 8.1.1, GraphPad Software, San Diego, CA, USA). *P* ≤ 0.05 was considered statistically significant.
